# New Insights on Juvenile Psoriatic Arthritis

**DOI:** 10.3389/fped.2022.884727

**Published:** 2022-05-26

**Authors:** Francesco Brunello, Francesca Tirelli, Luca Pegoraro, Filippo Dell'Apa, Alessandra Alfisi, Giulia Calzamatta, Camilla Folisi, Francesco Zulian

**Affiliations:** Department of Woman's and Child's Health, University of Padova, Padova, Italy

**Keywords:** juvenile psoriatic arthritis, children, diagnostic criteria, pathogenesis, treatment

## Abstract

Juvenile psoriatic arthritis (JPsA) is a relatively rare condition in childhood as it represents approximately 5% of the whole Juvenile Idiopathic Arthritis (JIA) population. According to International League of Associations of Rheumatology (ILAR) classification, JPsA is defined by the association of arthritis and psoriasis or, in the absence of typical psoriatic lesions, with at least two of the following: dactylitis, nail pitting, onycholysis or family history of psoriasis in a first-degree relative. However, recent studies have shown that this classification system could conceal more homogeneous subgroups of patients differing by age of onset, clinical characteristics and prognosis. Little is known about genetic factors and pathogenetic mechanisms which distinguish JPsA from other JIA subtypes or from isolated psoriasis without joint involvement, especially in the pediatric population. Specific clinical trials testing the efficacy of biological agents are lacking for JPsA, while in recent years novel therapeutic agents are emerging in adults. In this review, we summarize the clinical features and the current evidence on pathogenesis and therapeutic options for JPsA in order to provide a comprehensive overview on the clinical management of this complex and overlapping entity in childhood.

## Clinical Aspects

### Does JPsA Represent a Single Entity Within the JIA Spectrum?

Moll and Wright in 1971 defined psoriatic arthritis (PsA) as arthritis with psoriasis (1). Soon, this definition became too simplistic, making it necessary to create a new and more reliable definition, especially for the pediatric-onset forms (2). To date, the most widely used classification system is the International League of Associations of Rheumatology (ILAR) (3) that classifies JIA into seven different categories. According to ILAR, JPsA is defined by the association of arthritis and psoriasis or, in the absence of psoriasis, by at least two of the following: dactylitis, nail pitting, onycholysis or psoriasis in a first-degree relative. These criteria for JPsA replaced the less-restrictive Vancouver criteria, completely excluding from the JPsA group children with spondyloarthropathy and the ones with positive rheumatoid factor. These restrictions aimed to define a single diagnostic category for JPsA. However, while the ILAR classification is generally accepted for other forms of JIA, the differentiation into two non-overlapping diagnostic entities, enthesitis-related arthritis (ERA) and JPsA, has been subject of discussion (4). To date, a multicenter multinational study, conducted by the Pediatric Rheumatology INternational Trials Organization (PRINTO), is aimed to set up a new classification for chronic arthritis in children in order to identify more homogeneous entities, even comparing children with adults (5). For adults, indeed, ClASsification criteria for Psoriatic ARthritis (CASPAR) have been implemented more than a decade ago (6). These include feature of both peripheral and axial arthritis, as well as extra-articular manifestations, and demonstrate good diagnostic performance (7, 8). Interestingly, when applied to children, CASPAR allows to diagnose a larger number of JIA patients as having JPsA, compared to ILAR (9). As such, although some differences exist between adult and juvenile PsA, these criteria might be a useful starting point toward a more specific classification.

Part of the variability in JPsA reflects the divergent presentations in younger and older children. Since the first cases of arthritis and psoriasis in children were described, a bimodal presentation was found, demonstrating two peaks in the age of onset, one around 2 years of age, and a second in later childhood (10). In later years, the presence of two distinct clinical subgroups was confirmed in several studies, including also large cohorts of patients, such as those from the Childhood Arthritis and Rheumatology Research Alliance (CARRA) JIA registry (2, 9, 11, 12). Younger children have clinical features similar to early-onset oligoarticular and polyarticular JIA. Most of these patients are females, with anti-nuclear antibodies (ANA) positivity; association with Human Leukocyte Antigen (HLA)-DR5 is described (2, 5). On the other hand, older children with JPsA, typically adolescents, tend to develop enthesitis and features of spondyloarthritis, resembling adults with PsA (12). Both subgroups share some unique features that distinguish them from non-JPsA patients, including dactylitis and more frequent involvement of wrists and small joints (12). However, despite these clinical points of union, the overall impression is that, while younger children have phenotypic and pathophysiological features compatible with classical autoimmune diseases, the other subgroup, composed of older individuals, displays features of autoinflammation manifesting as enthesopathy (12).

### Which Are the Main Rheumatological Features of PsA in Children?

Arthritis in JPsA is often oligoarticular at onset but tends to extend to five or more joints in 60%−80% of patients in the absence of effective therapy. The most involved joints are knee and ankle. Wrists, ankles and small joints of hands and feet are affected more frequently than in other subtypes of oligoarthritis (13). Involvement of the distal interphalangeal joints is highly suggestive of JPsA, although rare at onset. JPsA can affect the axial skeleton in 10%−30% of patients: sacroiliitis, often asymmetrical, mainly affects patients with late disease onset, particularly those who express the HLA-B27 (11). Of note, HLA-B27 positivity is considered as an exclusion criterion for JPsA according to ILAR, thus limiting the proper classification of patients with spondyloarthitis. Indeed, up to 40% of children might rather fall under the “undifferentiated arthritis” group or other subtypes when this classification is applied (9, 14).

Enthesitis, the inflammation of the insertion of ligaments and tendons into a bone segment, is a hallmark of psoriatic arthritis in adults and is present in up to 60% of late onset JPsA, compared to only 22% of younger patients (15). Typical sites of enthesitis include the insertions of the Achille's tendon and plantar fascia in the calcaneus. Performing a joint and muscle-tendon ultrasound (MSUS) can be helpful in highlighting the inflammation of these entheses and is of great help in diagnosing JPsA.

Dactylitis is another clinical hallmark, which is present in 20%−40% of patients with JPsA and represents the only musculoskeletal finding at presentation in around 15% of them (16). Dactylitis is defined as swelling of a finger that extends beyond the joint limits. The swelling may be uniform along the length of the finger, giving the appearance of a “*sausage finger*,” but may also be fusiform with accentuation around the proximal interphalangeal joint. Adult studies suggest that dactylitis results from a variable combination of tenosynovitis of the flexors, synovitis in neighboring joints, growth of new subperiosteal bone and enthesitis at multiple insertions of tendons, ligaments and other fibrous structures that allow flexion of the fingers without formation of flexor tendon arches (17).

### Which Are the Dermatological Features of PsA in Children?

Psoriasis occurs in 40%−60% of patients with JPsA, usually the classic vulgaris form, although guttate psoriasis is also observed. Psoriasis in children tends to be subtle with thin, soft plaques that may be similar to atopic eczema (18). Onychopathy is reported in more than half of patients with JPsA, compared with 30% in childhood psoriasis in general. Onycholysis (separation of the nail from the nail bed) may also be observed, but is much less common than in adults. Interestingly, these nail changes may reflect enthesitis of the distal insertion of the extensor tendons, a site in intimate contact with the nail bed (19, 20).

### Which Are the Ophtalmological Features of PsA in Children?

Like in other forms of JIA, among extra-articular and extra-cutaneous complications, children with JPsA can manifest with uveitis. Painless chronic uveitis occurs in 10%−15% of children with JPsA and is indistinguishable from that seen in oligoarticular and polyarticular JIA. Young and ANA-positive patients appear to be at higher risk. Acute anterior uveitis is generally a manifestation of a subset of children with ERA who are HLA-B27 positive with ERA and in some patients with PsA (4, 21).

### Is PsA Different in Adults and in Children?

Psoriatic arthritis of adulthood is a well-defined, although phenotypically heterogeneous, clinical condition. In the majority of cases, it is characterized by the onset of arthritis in patients with pre-existing psoriasis. In adults, 20%−30% of psoriatic patients present joint involvement and arthritis onset manifests on average 6–7 years after the diagnosis of psoriasis (22, 23). Strikingly, an opposite scenario is seen in children: arthritis complicates only 2% of pediatric psoriasis (24), whereas in JPsA skin disease typically occurs up to 10 years after the development of arthritis, making JPsA diagnosis often challenging (25, 26). Overall, peripheral joint involvement is the most common presentation in both adult and juvenile PsA; axial arthritis can complicate both forms, but in JPsA in less common and generally milder than the adult counterpart. Oligoarticular ANA negative arthritis is generally more typical in adults, although the prevalence of oligo- and polyarticular presentation is quite variable (26, 27). In children, oligoarticular onset with evolution to extended/polyarticular is frequently reported. Regarding outcomes, joint involvement in PsA in often severe, with bone erosions and deformities in almost half of cases (23). In JPsA, Southwood et al. (25) in the 90's reported radiological bone damage in around 25% of patients (25). Another study from North America recently confirmed this finding despite the wider use of Disease Modifying Antirheumatic Drugs (DMARDs) (9), highlighting the severity of disease also in children. The more destructive form of PsA, known as *arthritis mutilans*, is however anecdotal in children (28).

As earlier described, enthesitis and dactylitis are hallmarks of PsA both in children and adults, with similar reported prevalence in both groups (26). Interestingly, a recent retrospective cohort study described the association between JPsA and other inflammatory conditions, highlighting a higher risk for developing Inflammatory Bowel Diseases (IBD), as described also in adults (24). Differences and similarities between PsA and JPsA are summarized in Table 1.

**Table 1:** Differences between adult and pediatric-onset psoriatic arthritis.

**Clinical feature**	Adult PsA*	**JPsA^†^**
Timing of psoriasis and arthritis onset	psoriasis prior to arthritis	arthritis prior to psoriasis
Peripheral arthritis		
•Oligoarticular	20%−55%	45%−55%
•Polyarticular	20%−60%	35%−55%
•Oligo-Extended		15%−38%
Axial arthritis	7%−40%	10%−30%
Radiological damage	47 %	25%
Enthesitis	30%−50%	12%−45%
Dactylitis	40%−50%	17%−37%
Nail involvement	41%−93%	37%−57%
Uveitis	8%	8%−13%
HLA-B27	40%−50%	10%−25%
ANA positive	16%	40%−46%

*PsA, Psoriatic Arthritis; JPsA, Juvenile Psoriatic Arthritis; HLA, Human Leukocyte antigen; ANA, Antinuclear Antibodies*.

* *Cumulative data from references (18, 19, 22, 23)*.

† *Cumulative data from references (8–10, 21)*.

## Pathogenesis

### What Is New About the Pathogenesis of PsA?

Given the paucity of studies for JPsA, pathogenic aspects of this condition can be mainly inferred from the adult counterpart. In this section, we summarize the evidence regarding genetic and immunologic aspects mainly of adult PsA. Recent studies focused on the search of clinical, genetic and molecular markers associated with the risk of developing PsA in patients with psoriasis (29). Despite the presence of phenotypic differences, family aggregation studies suggest a common genetic background between psoriasis and arthritis. These conditions share several susceptibility factors, including genes involved in immune-inflammatory responses and in epidermal differentiation (30). An important role is played by the major histocompatibility complex. The HLA-C06:02 allele has been described as the main susceptibility factor for both psoriasis and arthritis and, interestingly, it has been associated with treatment responsiveness. Recent studies, in fact, show that response to adalimumab and ustekinumab varies among positive or negative to HLA-C06:02 individuals. Particularly, HLA-C06:02 negative patients exhibit a greater response to adalimumab, while HLA-C06:02 positive patients to ustekinumab (31). Case-control studies comparing patients with psoriasis and those with PsA have shown that the HLA-B27, B38, B39 and B07 alleles are specifically associated to the PsA phenotype (32–35). B38 and B39 alleles are related to the peripheral polyarticular involvement, while HLA-B27 is mainly associated with axial involvement, enthesitis, dactylitis and uveitis. In addition, patients with PsA expressing HLA-B27 tend to have a shorter time interval between the onset of skin disease and first skeletal symptoms. Thus, although found in smaller percentage in patients with only skin disease, HLA-B27 represents one of the strongest HLA risk factors for PsA in patients with psoriasis and could help differentiate the two conditions (36).

As for the pediatric population, few data are available on genetic predisposition in JPsA. A higher prevalence of HLA-B27 allele among patients with JPsA has been described (37). Thomson et al. (38) also found an association between HLA class II genes (HLA-DRB1^*^01) and JPsA and this was independent from HLA-B27.

Other than the genetic background, the most recent studies focused on specific immunologic features in PsA. First, some histopathological features allow to differentiate it from other common types of arthritis. These include convoluted and immature vessels in the synovia, greater number of immature and constantly activated dendritic cells promoting a self-maintaining inflammatory state and a rich lymphocyte infiltrate (39).

As for circulating lymphocytes, CD4^+^/IL-17^+^ and CD4^+^/IL-22^+^ T-lymphocytes have been investigated with great interest because of their role in skin infection and in the skin of patients with psoriasis. The high efficacy of anti-interleukin (IL)-17 drugs in psoriasis confirmed the role of these lymphocytes in inducing chronic inflammation in the skin. Interestingly, studies in synovial fluid from patients with PsA have shown increased CD4^+^/IL-17^+^ T-lymphocytes and decreased CD4^+^/IL-22^+^ T-lymphocyte, suggesting their different role in the pathogenesis of PsA vs. psoriasis (40).

In light of the emerging association between PsA and defined HLA class-I haplotypes, studies have also explored the pathogenic role of CD8^+^ T-lymphocytes (41). Clonal expansion of CD8^+^/CXCR3^+^/ZNF683^+^ T-lymphocytes in the synovial fluid of patients with PsA suggest a relevant role of resident lymphocytes responses against certain antigens (42). Other Authors reported an increase in CD8^+^/CCR10^+^ T-lymphocytes in the peripheral blood of patients with PsA when compared to those with just psoriasis (43). These lymphocytes are characterized by a Tc2/22-like cytokine profile and have cutaneous homing. Thus, it is possible that the immune dysregulation, primarily affecting the cutaneous tissues, could cause an increase in circulating T-lymphocytes from the skin. These lymphocytes could then facilitate joint inflammation by releasing specific pro-inflammatory cytokines.

Overall, these studies collect the evolving picture of a complex pathogenesis in which the main point relies on the activation of a local immune microenvironment (joint) in response to a specific and still unidentified antigenic stimulus. This process may be supported by a predisposing genetic background and favored by a systemic inflammatory state, perhaps derived from a preexisting cutaneous immune dysregulation.

## Treatment

### What Is the Standard of Care in JPsA?

The American College of Rheumatology (ACR) recently updated the recommendations for JIA published in 2011 with the intent to provide guidance for treatment in non-systemic polyarthritis, sacroiliitis and enthesitis (44). It was decided to base the current guideline on broad clinical phenotypes rather than ILAR categories, since more recent data suggest that these categories may not entirely reflect the underlying genetic and clinical heterogeneity of the disease or be relevant for guiding treatment decisions (45).

As such, treatment strategies, especially first- and second-line steps, do not differ from those used also in other JIA categories, particularly those for polyarticular disease including JPsA. At the same time, there are some noteworthy differences to report in relation to distinct clinical and biochemical features of the disease.

Nonsteroid anti-inflammatory drugs and oral glucocorticoids, as well as intra-articular glucocorticoids, are indicated as initial steps for symptom relief and bridge therapies (46, 47). DMARDs represent the mainstay second line treatment of children with polyarthritis (44). The most used is methotrexate (MTX), which is recommended over leflunomide or sulfasalazine (48, 49). Methotrexate is usually well tolerated in children, while it is associated with an increased risk of hepatotoxicity in adults with PsA compared to those with Rheumatoid Arthritis (RA) (50, 51).

As indicated by ACR recommendations, biologic agents should be considered in case of DMARDs failure or intolerance, presence of risk factors or high disease activity (44).

Among tumor necrosis factor inhibitors (TNFi), which are commonly used in many JIA categories, the only molecule whose indications are extended to JPsA is Etanercept (ETN), a biologic fusion protein that binds circulating TNF-alpha, avoiding its interaction with cell receptors and thus the propagation of inflammation (52). As to Food and Drug Administration (FDA) and European Medicines Agency (EMA), it is indicated for children aged 12 and over with JPsA or ERA with insufficient response to conventional therapy. ETN has been shown effective in several large cohorts of PsA patients and also shows the best cost-effectiveness profile among biologics (53–55). Prospective evidence to support its use in children has been provided by the CLIPPER study. In this open-label trial, which included three subtypes of JIA, JPsA patients showed significant improvement in disease activity and a favorable safety profile over 2 and 6 years (56, 57). Several other TNFi are approved and recommended in adult PsA (23, 58, 59), such as infliximab, golimumab, certolizumab pegol and adalimumab, the latter also approved for juvenile psoriasis; however, specific data on their effectiveness in JPsA remain scarce.

### Which Are the New Therapeutic Agents for JPsA in the Pipeline?

Novel agents are becoming increasingly available for the treatment of chronic inflammatory arthritides, expanding the therapeutic options for pediatric and adult patients.

Synthetic DMARDs, such as Janus Kinase (JAK) inhibitors, are emerging as promising treatments for adult PsA (60, 61); recently, tofacitinib, already used for adults, has been approved for JIA and JPsA (62).

Development of novel biologic agents focuses on targeting specifical pathogenic molecular cascades. As previously stated, IL-17 plays a pivotal role in psoriatic skin and joint inflammation (40); thus, blockade of IL-17 pathway, including regulatory cytokines IL-12 and IL-23, raises particular interest for the treatment of JPsA (Table 2).

**Table 2:** Biological agents approved or currently under study for JPI sA.

**Biologic agent**	**Target**	**Role in pathogenesis**	**Availability for JPsA**
**Etanercept**	TNF alpha	Proinflammatory cytokine, found elevated in skin and synovial fluid in psoriasis and PsA (19).	Approved by FDA and EMA for JPsA ≥12 year-old patient.
**Tofacitinib**	JAK1 JAK3	JAK/STAT pathway is involved in the inflammatory cascade induced by several cytokines, including IL-12, IL-23, TNF alpha.	Approved by FDA and EMA for JPsA ≥2 year-old patients.
**Secukinumab**/ **Ixekizumab**	IL-17A	Main proinflammatory cytokine produced by Th17 lymphocytes, which plays a crucial role in skin and synovium inflammation in PsA (23, 40).	Secukinumab: ongoing phase III study for active JPsA or ERA.
			Ixekizumab: approved for juvenile plaque psoriasis. Currently under investigation for juvenile SPA
**Ustekinumab**	P40 subunit of IL-12 and IL-23	IL-23 induces differentiation of Th17 cells and production of IL-17, main pathogenic cytokine in PsA (19, 35).	Ongoing trial for juvenile refractory psoriasis in adolescents. No prospective studies for JPsA.
**Guselkumab**/ **Rizankizumab**	IL-23	IL-23 induces differentiation of Th17 cells and production of IL-17, main pathogenic cytokine in PsA (19, 35).	Approved by FDA and EMA for adult psoriasis and PsA. Ongoing trials for pediatric psoriasis.

*PsA, psoriatic arthritis; JPsA, juvenile psoriatic arthritis; IL, interleukin; JAK, janus kinase; STAT, signal transducer and activator of transcription; TNF, tumor necrosis factor; ERA, enthesitis–related arthritis; SPA, spondyloarthropathy; FDA, Food and Drug Administration; EMA, European Medicines Agency*.

Secukinumab (Anti-IL-17) is a monoclonal antibody that binds IL-17A, thus blocking the interaction with its cellular receptor and the subsequent inflammatory cascade (63). Secukinumab is actually approved by EMA in adults for refractory PsA and ankylosing spondylitis. A double-blind, placebo-controlled, randomized, multicenter study to evaluate the efficacy of secukinumab in active JPsA or ERA is ongoing and very promising (ClinicalTrials.gov identifier: NCT03031782). Another monoclonal antibody against IL-17A, ixekizumab, is approved for the treatment of adult and juvenile plaque psoriasis and for refractory PsA. No data are available on the use of ixekizumab for JPsA, but this agent is being investigated in a prospective trial for its use also in juvenile spondyloarthropathies (ClinicalTrials.gov identifier: NCT04527380).

Ustekinumab (Anti-IL-12/23) is a fully human monoclonal antibody binding the p40 subunit of IL-12 and IL-23, preventing their interaction with IL-12 receptor and thus immune cells activation. Current indications, as to EMA, are for the treatment of adults with refractory IBD, while promising results have been shown also for psoriasis and PsA in adults (64). In children, some case reports confirmed the efficacy of ustekinumab in psoriasis, JpsA and IBD (65–70). An ongoing phase III randomized, double-blind, placebo-controlled, multicenter study (CADMUS) aims to evaluate safety and efficacy of ustekinumab in adolescents with moderate to severe psoriasis (ClinicalTrials.gov identifier: NCT01090427).

Interestingly, IL-23 inhibitors include other agents initially used for psoriasis, such as guselkumab and risankizumab, that, following recent trials, showed efficacy and are now approved for adult PsA (71–75). Although few data are available for pediatric patients, this opens other future possibilities for juvenile psoriasis and JPsA.

We are looking forward to seeing the results of the ongoing trials in the next future, especially for the treatment of refractory JPsA.

## Conclusion

In summary, JPsA is an entity characterized by distinct clinical features (dactylitis, small joints involvement, skin involvement) but with a broad phenotypic spectrum, which sometimes overlaps with other forms of JIA. Specific features, such as ANA in younger females or HLA-B27 positivity, correlate with specific phenotypes and should therefore be considered to guide clinical characterization and therapeutic choices. Defining more homogeneous entities, along with further understanding of disease pathogenesis, could be crucial to successfully extend to the pediatric population the most recent biological therapies approved for adults with PsA.

## Author Contributions

FB: study concept and design, literature review, and drafting and revision of the manuscript. FT: study concept and design, literature and references review, drafting and revision of the manuscript, and study supervision. LP, FD'A, AA, GC, and CF: study design, literature review, and drafting of the manuscript. FZ: study concept and design, critical revision of the manuscript, and study supervision. All authors contributed to the article and approved the submitted version.

## Conflict of Interest

The authors declare that the research was conducted in the absence of any commercial or financial relationships that could be construed as a potential conflict of interest.

## Publisher's Note

All claims expressed in this article are solely those of the authors and do not necessarily represent those of their affiliated organizations, or those of the publisher, the editors and the reviewers. Any product that may be evaluated in this article, or claim that may be made by its manufacturer, is not guaranteed or endorsed by the publisher.
